# The vicious spiral in Sudden Infant Death Syndrome

**DOI:** 10.3389/fped.2025.1487000

**Published:** 2025-02-11

**Authors:** Siri Hauge Opdal, Arne Stray-Pedersen, Johanna Marie Lundesgaard Eidahl, Åshild Vege, Linda Ferrante, Torleiv Ole Rognum

**Affiliations:** ^1^ Section of Forensic Research, Department of Forensic Sciences, Oslo University Hospital, Oslo, Norway; ^2^ Section of Forensic Pathology and Forensic Clinical Medicine, Department of Forensic Sciences, Oslo University Hospital, Oslo, Norway; ^3^ Department of Forensic Medicine, Institute of Clinical Medicine, University of Oslo, Oslo, Norway

**Keywords:** hypoxia, infection, risk factors, serotonergic network, SIDS, sudden infant death, triple risk hypothesis of SIDS, vicious spiral of SIDS

## Abstract

Sudden Infant Death Syndrome (SIDS) is the sudden and unexpected death of an otherwise healthy infant less than 1 year of age where the cause of death remains unexplained after a thorough post-mortem investigation and evaluation of the circumstances. Epidemiological, clinical, biochemical, immunological and pathological evidence indicates that three factors must coincide for SIDS to occur: a vulnerable developmental stage of the immune system and central nervous system in the infant, predisposing factors, and external trigger events. This model is referred to as the fatal triangle or triple risk hypothesis. The concept of a vicious spiral in SIDS, starting with the fatal triangle and ending in death, is proposed as a model to understand the death mechanism. The vicious spiral is initiated by a mucosal infection and immune activation in the upper respiratory and digestive tracts, increased production of cytokines, and an overstimulation of the immature and rapidly developing immune system. A second trigger is the prone sleeping position, which may lead to rebreathing and hypercapnia, in addition to intensify the immune stimulation. In susceptible infants, this induces an aberrant cytokine production that affects sleep regulation, induces hyperthermia, and disrupts arousal mechanisms. In turn, this initiates downregulation of respiration and hypoxemia, which is worsened by nicotine. Inefficient autoresuscitation results in severe hypoxia and accumulation of hypoxic markers which, if not prevented by a normally functioning serotonergic network, contribute to a self-amplifying vicious spiral that eventually leads to coma and death. The purpose of this review is to summarize the research that underpins the concept of the vicious spiral.

## 1 SIDS definition

The first definition of Sudden Infant Death Syndrome (SIDS) was established during “The Second international conference on causes of sudden death in infants” in Seattle in 1969: “The sudden death of any infant or young child which is unexpected by history, and in which a thorough post-mortem examination fails to demonstrate an adequate cause of death” (1). This was followed by a slightly revised definition in 1991, the National Institute of Child Health and Human Development (NICHD) definition, where an investigation of the death scene and also review of the clinical history was included: “The sudden death of an infant under one year of age which remains unexplained after a thorough case investigation, including performance of a complete autopsy, examination of the death scene, and review of the clinical history” (2).

The current commonly used SIDS definition is from 2004, now including an expanded general definition of SIDS and further subdivision into different SIDS categories (IA, IB, or II), depending upon the amount of information available (3). Commonly referred to as the San Diego definition, the definition says “SIDS is defined as the sudden unexpected death of an infant <1 year of age, with onset of the fatal episode apparently occurring during sleep, that remains unexplained after a thorough investigation, including performance of a complete autopsy and review of the circumstances of death and the clinical history”. This definition only includes infants <1 year of age, which is why an additional definition for Sudden Unexplained Death in early Childhood (SUDC) has been proposed (4). According to this, SUDC is defined as “The sudden death of a child older than one year of age which remains unexplained after a thorough case investigation, including review of the clinical history and circumstances of death, and performance of a complete autopsy with appropriate ancillary testing”.

Despite efforts to provide international standards on how to perform autopsy on infants, including ancillary testing and how to interpret findings, there seems to be a lack of consensus among pathologists and death certifiers on how to classify unexplained infant deaths (5–7). In most countries the reduction in SIDS rate follows in parallel the decrease in total post neonatal mortality (8). To a certain extent this reduction may have been exaggerated by the attribution of these deaths to causes, like positional asphyxia (9). A recent study showed that unexplained infant deaths in the US are now more often designated “undetermined” or due to “accidental suffocation” than classified as SIDS (10). Lack of a generally accepted diagnosis for sudden unexpected and unexplained deaths in infants and children hinders effective surveillance, prevention and research (11). Motivated by the increasing rejection of the SIDS diagnosis, an initiative has been taken to develop a unified diagnosis for sudden death in paediatrics which might be accepted by the World Health Organization for inclusion in the upcoming ICD-11. A broader definition of the SIDS diagnosis will most likely include all sudden unexpected deaths not fully explained after a thorough post-mortem investigation (11).

## 2 The triple risk hypothesis in SIDS

The first version of a triple risk hypothesis for SIDS was introduced by Wedgwood in 1972 (12). He postulated three elements that are involved in these deaths: (1) general risk factors such as low socioeconomic status and sex, (2) age-dependent factors relating to the developmental stage of the infant, including immunological status and the changing physiological responsiveness with regard to cardiopulmonary reflex control, and (3) external stimuli, including sleeping position and viral infections. A few years later Valdés-Dapena proposed a two-hit model for SIDS; a previous hypoxic episode, followed by a second fatal event (13, 14). Her model was based on the studies by Naeye et al., who identified various tissue-markers for hypoxia and hypoxemia, as well as her own observations in SIDS victims (15).

**Figure 1:**
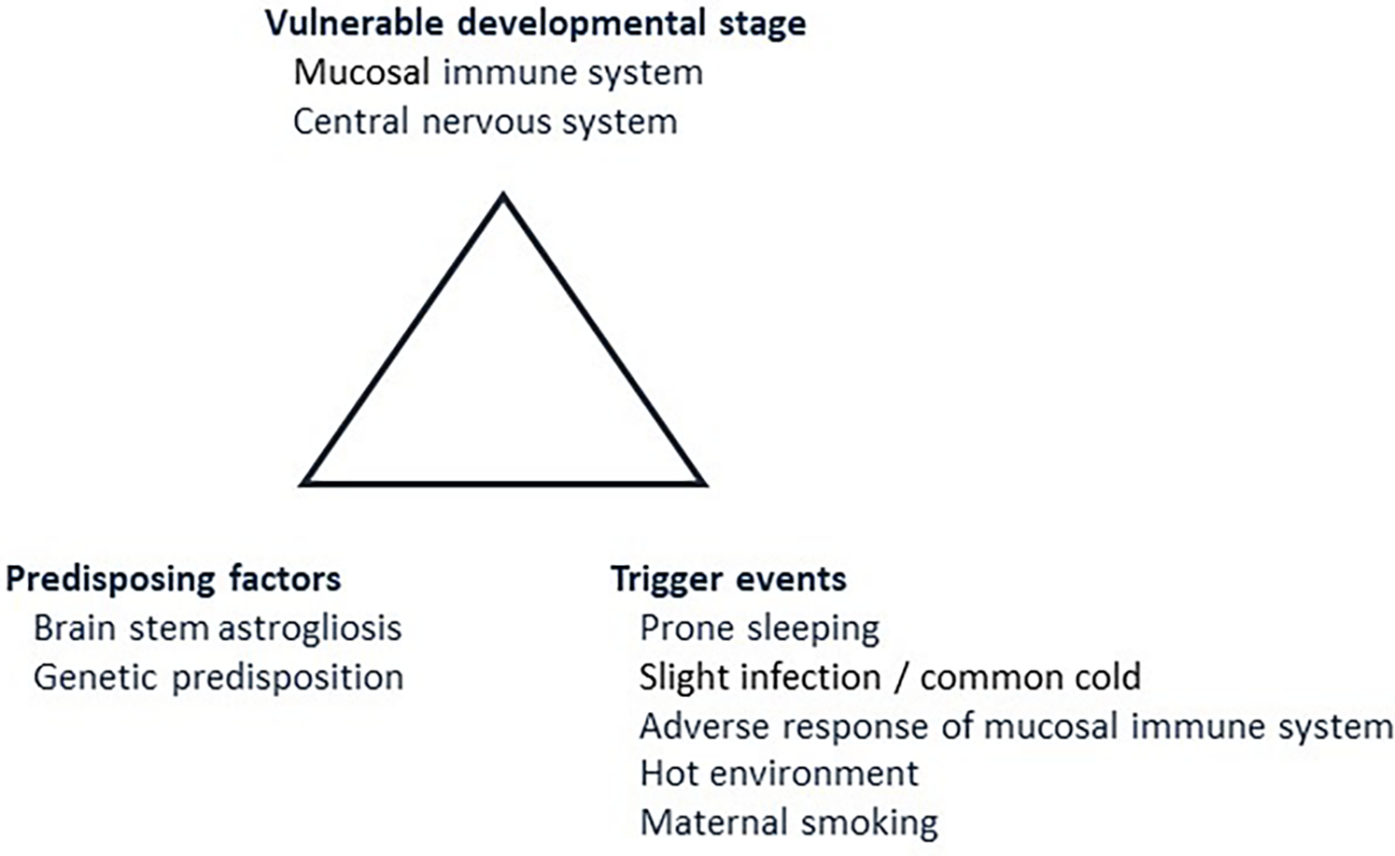
The fatal triangle in SIDS, modified after Rognum and Saugstad (16). This model postulates that SIDS occurs when an infant is at vulnerable developmental stage in maturation of the immune system and central nervous system (2.1). At the same time, predisposing factors, such as brainstem astrogliosis and/or predisposing gene variants must be present (2.2). Lastly, the infant must be exposed to external trigger events such as prone sleeping, slight infection, for example a common cold, and/or maternal smoking (2.3). Numbers in brackets refer to paragraphs in the text.

In 1993, Rognum and Saugstad introduced the concept of a “fatal triangle” in SIDS, which was based on morphological, biochemical and immunological findings (Figure 1) (16). They demonstrated elevated levels of the hypoxic marker hypoxanthine in SIDS compared to violent infant death (17, 18), and also increased immune reaction in the upper airways and digestive tract in SIDS compared to infants dying of non-infectious causes (19, 20). Recognizing the typical age peek for SIDS between 1 and 6 months of age and the coinciding rapid development of mucosal immunity during this period prompted the introduction of a vulnerable development stage (21, 22). A possible link between the peripheral immune system and the central nervous system was based on the observation that SIDS victims often exhibited signs of infection prior to death, manifested immune responses in the laryngeal mucosa, and displayed elevated levels of interleukin 6 (IL-6) in the cerebrospinal fluid (23, 24). It was thus postulated a fatal tringle in SIDS. This consists of a vulnerable developmental stage, predisposing factors, including a genetic predisposition, and trigger events such as a stimulation of the mucosal immune system due to a common cold (Figure 1) (16).

Filiano and Kinney presented another and slightly different version of the triple risk model (25, 26). According to this model, SIDS occurs when three factors impinge upon the infant simultaneously: an underlying vulnerability in protective responses to hypoxia, a critical developmental phase in homeostatic control, and exogenous stressors that induce life-threatening hypoxia, such as prone or facedown sleep position. The vulnerability remains dormant until the infant reaches a critical stage of development and is exposed to a stressor, such as unsafe sleeping practices like prone sleeping, co-sleeping, excessive bedding, or passive smoking. The underlying vulnerability or abnormality in the brainstem in SIDS victims differentiates them from healthy infants; in the latter, protective brainstem responses are activated when exposed to homeostatic challenges.

The following sections will elaborate the fatal triangle in more detail (Figure 1).

### 2.1 Vulnerable developmental stage

The fatal triangle in SIDS suggests an age-dependent increased risk for sudden death in infancy. During the first months of life, there is a rapid development of both the central nervous system and the immune system, particularly in terms of homeostatic control, as the infant adapts to the microbial environment outside the womb. This period is recognized as a vulnerable developmental stage. Within the central nervous system, significant changes occur in the serotonergic network, which is crucial for chemo-sensitivity, respiration, upper airway reflexes, blood pressure, thermoregulation and arousal (27, 28). Simultaneously, the infant's mucosal immune system undergoes rapid development, making the infant more susceptible to various immune stimuli (21, 22, 29, 30). IgG from the mother disappears quickly. At the same time, the secretory immune system undergoes a rapid development, and the number of IgM and IgA immunocytes, as well as the transport of IgM and IgA by secretory component through the epithelia increase dramatically from the second week after birth (30, 31). It is reasonable that disappearance of maternal IgG and the rapid development of the immune system in the period from the fourth postpartum week makes infants vulnerable for unfortunate and possibly dangerous immune reactions. This suspicion has been strengthened by the observation of enhanced immune responses in the respiratory and gastrointestinal tract in SIDS (19, 32, 33). These observations are supported in a prospective study of immunoglobulins and albumin in saliva sampled repeatedly in 263 infants from day two until eight weeks postpartum (30). One infant in the cohort died from SIDS at the age of eight weeks. Whereas saliva from the cohort showed a relative rapid increase of IgA and IgM from the second week after birth, the SIDS victim experienced a much steeper increase of both IgA and IgM from the fourth week of life. At week eight, the SIDS case had reached a about a nine-fold increase in IgA concentration and a six-fold increase in concentration of IgM, compared to the median in the controls (30).

Increased levels of IL-6 in the cerebrospinal fluid have been found in a large proportion of SIDS victims, and a relationship with immune responses in the respiratory tract has been demonstrated (23, 24, 34). These studies indicate that IL-6 may constitute a link between the mucosal immune system and the central nervous system. During infection, peripherally produced cytokines may cross the blood-brain barrier, either by retrograde axonal transport or by blood born, and bind to endogenous receptors on neuronal populations that mediate stress responses in the hypothalamus and/or brainstem, and thereby determine sickness behavior including fever, blunted arousal and depressed respiration (35, 36). A study from Harvard Medical School on samples from Norwegian SIDS victims disclosed abnormal IL-6R expression in the arcuate nucleus in the SIDS cases, of which 44% had signs of slight infection immediately prior to death (37). This observation seems interesting, since the reduction in SIDS rates in Norway to a large extent is due to less small infants with a history of common cold in the days before they were found dead in prone position (38).

Temporal shift in innate immunity may also contribute to increased vulnerability during the first months after birth. It is known that Surfactant protein A, which plays a role as an opsonic in the lungs by tagging foreign pathogens for elimination by phagocytes, substantially decreases in infants between 1 and 5 months of age (39). This further emphasizes the vulnerability in the immune system during this period.

### 2.2 Predisposing factors

Multiple predisposing factors contribute to SIDS, including brainstem astrogliosis, neurochemical imbalances in the serotonergic network, and genetic predispositions (Figure 1).

Brainstem astrogliosis may develop as a nonspecific response to injury in the central nervous system as early as in the second trimester of a pregnancy. Studies have reported an increased number of reactive astrocytes in the brainstem in SIDS compared to controls, with a correlation observed between brainstem astrogliosis and maternal smoking during pregnancy (15, 40, 41). Gliosis typically takes 3–4 days to develop, suggesting it is not a consequence of events immediately preceding death but rather linked to an injury occurring at least a few days prior to death.

Compelling evidence implicates the serotonergic network in SIDS, although the question remains whether serotonergic abnormalities originate from prenatal or postnatal development (26, 42). Several findings support a developmental origin, such as immature serotonergic neurons in the medulla, decreased serotonergic receptor binding in the arcuate nucleus in SIDS, and abnormal expression pattern of proteins crucial for neurological development (43, 44).

The genetic predisposition most likely represents a polygenic inheritance pattern; a combination of various gene variants and polymorphisms that collectively increase susceptibility. This genetic predisposition remains dormant and inconsequential until the infant reaches a critical developmental stage and coincides with exposure to external risk factors known to increase the risk for sudden death in infancy.

Over the years, numerous genes and gene regions have emerged as important in the context of SIDS (45–49). Initial investigations focused on genes involved in the function and regulation of the immune system, including complement component C4 and different cytokines and interleukins (50–52). Furthermore, genes associated with brain development and function are likely to contribute to the genetic predisposition for SIDS. The most important genes in this category include genes involved in the serotonergic network and genes encoding different ion channels and aquaporins (53–61). Another crucial area involves genes related to cardiac function and arrhythmia (62–65).

It is crucial, particularly concerning cardiac genes, to distinguish between gene variants that may constitute a predisposition for SIDS in specific contexts and pathogenic gene variants confirmed to induce arrhythmias and possibly death. When genetic heart disease is identified, the cause of death is clarified, and the case is no longer classified as SIDS.

### 2.3 Trigger events

The concept of the fatal triangle and triple risk hypotheses represent classical SIDS theories, where trigger events and risk factors are circumstances that may be demanding for an already vulnerable infant (66). It is important to note that trigger events are not the direct cause of death but rather initiators of a chain of reactions leading to SIDS.

Several of the trigger events are associated with sleeping environment. These include prone sleeping position and hazardous sleeping situations such as soft bedding, head covering, excessive clothing, and unsafe bed sharing (67–69). Public health campaigns promoting supine sleeping began in the early 1990s and significantly contributed to the decline in the SIDS rates (70). The prone sleeping position with face down, soft bedding and bed-sharing may all affect the infant's breathing, leading to rebreathing and thus hypercapnia. In unfortunate cases, the sleeping position may obstruct the airways, leading to positional asphyxia. Such cases should not be misclassified as SIDS. A thorough post-mortem examination, including a detailed investigation of the death scene, preferably with doll reenactment, makes it possible to rule out accidental suffocation as a cause of death in most cases (71). The increased risk for sudden infant death posed by prone sleeping position with face down and environmental risk factors cannot solely be attributed to mechanical breathing obstruction (72, 73).

Multiple studies have demonstrated that both pre- and postnatal nicotine exposure increases the risk of SIDS in a dose-dependent manner (74, 75). While the highest risk is associated with maternal smoking, especially during pregnancy, there is also a small independent risk associated with paternal smoking after the infant's birth (76, 77). A significant association has also been highlighted between smoking exposure and the biochemical lack of detoxification enzymes (78). The risk is further increased in the presence of additional risk factors, such as co-sleeping or prone sleeping combined with an infection (67, 79, 80). Mild upper airway infection during the last week or days prior to death was observed in more than half of the SIDS cases, with around 10%–15% exhibiting fever during the same period (67, 79). Interestingly, the dramatic decrease in SIDS rates in Norway from the 1980s to the 1990s was largely attributed to fewer SIDS in the age group 2–4 months with the combination of prone sleeping position and minor infection prior to death (38).

The overall SIDS mortality rate has decreased during the last three decades, in concordance with overall postnatal mortality (8, 81). The decreased SIDS rate is largely due to back-to-sleep campaigns and reduced rate of maternal smoking, although improvements in perinatal healthcare and infant sleep environment also may have had substantial impact (8, 81).

## 3 The vicious spiral of SIDS

As early as 1973, Beckwith proposed that SIDS represents a final common pathway in which similar agonal mechanisms of death are shared by the majority of cases (82). In 1999, Vege and Rognum proposed that a potential death mechanism in classical SIDS could be thought of as a vicious circle, starting with the fatal triangle and ending with death (83, 84). A similar sequence of events, focusing on failures in protective mechanisms against life-threatening events during sleep, was proposed by Kinney and Thach in 2009 (85).

**Figure 2:**
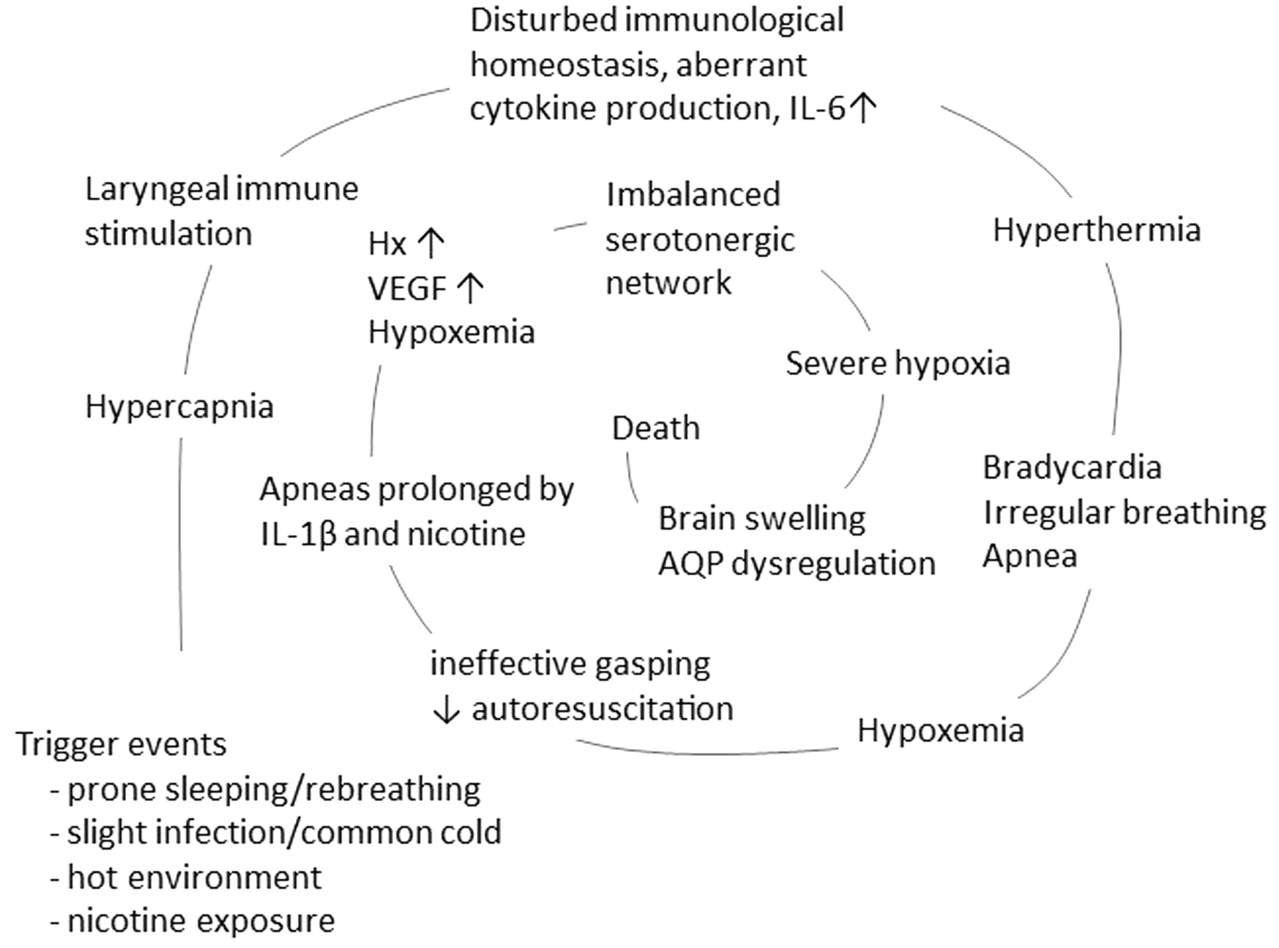
The concept of a vicious spiral of SIDS, suggesting a final common pathway in the majority of SIDS, adapted from Vege et al. 1999 and 2004 (83, 84). The model propose that in the vulnerable infant, a combination of triggers, such as prone position, hot environment and minor infection/common cold, may initiate a spiral leading to death.

The vicious spiral is initiated by a mucosal infection causing immune activation in the upper respiratory and digestive tract, increased production of cytokines, and overstimulation of the rapidly developing immune system. In susceptible infants, this may trigger aberrant cytokine production or a cytokine storm, which, via retrograde axonal transport, affects sleep regulation and arousal. This, in turn, initiates downregulation of respiration and accumulation of hypoxic markers which is not counteracted by rescue mechanisms, resulting in a vicious spiral that ultimately results in death (Figure 2).

Each step of the vicious spiral will be elaborated in the following sections.

### 3.1 Hypercapnia

The synergistic effect of hazardous sleeping conditions and infection can lead to hypercapnia. Prone sleeping or sleeping in a crib crowded with bedclothes or stuffed animals facilitate rebreathing and increased CO_2_ level (72). Additionally, even mild upper respiratory infection can increase metabolic rate and consequently CO_2_ levels in infants older than 3 months (86, 87). Furthermore, hypercapnia have the ability to increase the expression of cytokines and interleukins, including interleukin 1β (IL-1β), tumor necrosis factor α (TNF-α) and IL-6 (88, 89). A possible pathophysiological mechanism initiating the vicious spiral could be the combination of elevated CO_2_ levels and minor infection, triggering cytokine production and subsequent steps leading to death.

### 3.2 Infection and the immune system

A significant proportion of infants dying in SIDS exhibits signs of infection prior to death, and several risk factors associated with susceptibility to infection have been identified as risk factors for SIDS (84, 90, 91). In a Norwegian SIDS study, it was reported that 46% of the cases had a cold during their last week, and 33% had a cold on the day before death (67). Several studies report findings of bacteria in SIDS, and the common bacterial toxin hypothesis suggests that toxins from common bacteria could be a link between inflammation and SIDS (90–94). Studies have highlighted higher prevalence of *Staphylococcus aureus* in nasopharyngeal flora from SIDS, as well as staphylococcal endotoxins being more prevalent in samples from intestinal tract from SIDS compared to those from healthy controls (92, 94). Toxic shock syndrome and sepsis may develop rapidly from *S. aureus* or group A streptococcal infection and cause sudden death. The diagnosis of an infectious cause, rather than SIDS, relies on detection of bacteria in blood or cerebrospinal fluid, as limited pathological signs of inflammation may be detected in the organs with conventional histological methods. Post mortal bacterial growth of uncertain significance is common in samples taken after death, often attributed to post mortal growth. While some bacteria are well established as triggering pathogens and fatal infections, others are not.

*Helicobacter pylori* infection in childhood is associated with gastrointestinal disease but is mostly asymptomatic (95). However, a relatively large proportion of newborns have *H. pylori* antigen in stool (96, 97). The same was true for fecal specimen in a high proportion of SIDS victims and infants who died due to severe infection with other microorganisms (97). Interestingly, signs of *H. pylori* colonization were rarely present in infants who died of violent deaths or due to non-infectious diseases or malformations. The study hypothesized that *H. pylori* infection in infancy may be involved as a triggering pathogen for sudden death during the first 5 months after birth (97).

Viruses may also affect and compromise the immune response. Respiratory virus RNA has been detected more often in SIDS victims than in non-SIDS cases (98, 99). The seasonal variation involving a winter peak is observed both for virus epidemics causing respiratory infections and SIDS (100). Higher rates of herpesviruses, such as cytomegalovirus and Epstein-Barr virus have been reported in SIDS compared to controls, suggesting a relation and possible contribution in SIDS (101, 102).

Several studies have reported an activation of the mucosal immune system in SIDS. Both a higher number of IgM immunocytes in the tracheal wall, increased IgA immunocytes in the duodenal mucosa, and elevated IgG and IgA immunocyte density in the palatine tonsillar compartments have been reported in SIDS compared to controls (19, 32, 33, 103). In salivary glands an increased number of CD45^+^ stromal leucocytes, intensified expression of HLA-DR, and increased expression of HLA class I and II have been reported in SIDS compared to controls (20).

Numerous studies have investigated cytokines and interleukins in SIDS (23, 104–107). Initial studies examining IL-6 levels in the cerebrospinal fluid in SIDS disclosed elevated levels compared to controls, with about half of the SIDS victims exhibiting IL-6 levels within the same range as infants dying from severe infection (23, 104). SIDS cases with high IL-6 levels in cerebrospinal fluid and symptoms of mild infection prior to death also showed increased expression of both IgA and HLA-DR in the laryngeal mucosa (24, 34). Another interesting finding is the reported downregulation of myeloid differentiation primary response gene 88 (Myd88) in cerebral tissue from SIDS (108). Myd88 is crucial for both innate and adaptive immune responses, acting as a signal transducer in pathways that regulates the activation of several pro-inflammatory genes. Myd88 plays a pivotal role in initiating and sustaining an effective immune response. A deficiency in this protein may impair the ability to mount an optimal immune reaction, and potentially contributing to vulnerability in SIDS.

In summary, these findings suggest a potential common feature in SIDS; a faulty immunological response to an apparently mild infection (109), initiating an immune reaction in the laryngeal and tracheal mucosa, which either through retrograde axonal transport or by blood borne signals induce an elevated cytokine production within the central nervous system (24, 109, 110). IL-6 induces fever and deeper sleep, and interacts with the serotonergic network in the medulla oblongata (37). This interaction between the immune system and the serotonergic network may interfere with the homeostatic control of cardiorespiratory and arousal responses (26, 37).

### 3.3 Hyperthermia

Thermal stress and hyperthermia are frequently observed in SIDS. The hyperthermia can result from various external factors such as excessive clothing and high ambient temperature, or intrinsic factors such as mild infection (111, 112). Several studies have documented SIDS victims to be overdressed and/or are discovered in environments with elevated temperatures. Some are described as unusually warm or sweating when found dead (111, 113–115). The prone sleeping position potentiates the risk of overheating by reducing the surface area available for radiant heat loss. One study indicate that the heat loss coefficient can be up to 60% lower in a prone position compared to sleeping supine or on the side (116). In heavily wrapped infants sleeping prone, a combination of infection and a warm environment may be particularly harmful (117).

Thermal stress and overheating can significantly affect respiratory control; even mild thermal stress may potentially lead to an unstable breathing pattern (118). In 12-day-old mouse pups, hyperthermia enhanced the magnitude of bradycardia, suggesting that thermal stress may induce spontaneous bradycardia in an age dependent manner (119). Furthermore, prone sleeping position and subsequent reduced heat loss influence heart rate and blood pressure regulation, especially during the critical age range of 2–3 months when the risk of SIDS is highest (120). High ambient temperature (24°–28°C) could also impede arousal from REM sleep, particularly in the late hours of the night when most SIDS deaths occur (121).

### 3.4 Bradycardia, irregular breathing and apnea

Infection has been demonstrated to disrupt respiratory control mechanisms, alter breathing pattern, and impair arousal (122, 123). Both viruses and bacteria can provoke respiratory disturbances. In infants with respiratory syncytial virus (RSV) infection, it is shown that laryngeal stimulation results in disturbances in the regulation of breathing, most likely through the effect of IL-1β (124). IL-1β has also been found to induce prolonged apnea and modify autoresuscitation in piglets, and even low doses of nicotine and endotoxin have adverse effects on autoresuscitation following apnea by intensifying the effect of IL-1β (125–127).

High IL-1β immunoreactivity have been reported in the brainstem in SIDS (105). This may contribute to molecular interactions causing disturbed homeostatic control of cardiorespiratory and arousal responses. In addition, IL-10 may also play a role. The presence of IL-10 receptors on microglia suggests that IL-10 may affect the control of breathing since microglia modulate respiratory rhythm generation, breathing pattern, and autoresuscitation (128–130). Elevated levels of IL-10 have been reported in thymic tissue from SIDS (107). Another interesting finding, regarding nicotine exposure as a risk factor for SIDS, is that smokers are reported to have lower IL-10 production compared to non-smokers when exposed to infectious agents (131, 132).

An early polysomnographic study of infants who subsequently died of SIDS reported that obstructive and mixed apneas were more frequent and lasted longer in the SIDS victims compared to the control group (133). The study also observed a reduced number of sighs followed by apnea, suggesting a lower peripheral chemoreceptor response in some SIDS cases.

### 3.5 Hypoxemia and impaired arousal

Irregular breathing and apnea may lead to hypoxemia and subsequently hypoxia. In most infants this trigger gasping as a mechanism of autoresuscitation to restore tissue oxygenation. However, in SIDS, several studies have identified incomplete and less frequent arousal from sleep in response to hypoxia, indicating impaired autoresuscitation and a failure of reflexes that are supposed to be protective (134–136). This compromised autoresuscitation fails to restore the respiration, ultimately leading to severe hypoxia, coma and death.

It is notable that several significant risk factors for SIDS, including prone sleeping position, maternal smoking, prematurity and recent infection, all diminish infants’ ability to be aroused from sleep (137). Prone sleeping and maternal smoking have been shown to impair both stimulus-induced and spontaneous arousal, while sleeping in the prone position significantly impairs arousal from both active sleep and quiet sleep in healthy term infants at the age when SIDS incidence is highest (138–141).

A biochemical indication supporting impaired arousal in SIDS is the finding of a reduced orexin immunoreactivity in hypothalamic tissue in SIDS compared to controls (142). Orexin is a neuropeptide involved in respiratory control during sleep and in regulation of the sleep cycle. It has been suggested that hypoxic events occurring before death may be responsible for the decreased orexin immunoreactivity observed in cases of SIDS (142).

### 3.6 Hypoxia

Impaired autoresuscitation leads to hypoxemia and if no other rescue mechanisms are activated, it progresses to hypoxia. There are several biochemical markers of acute hypoxia, including hypoxanthine (Hx) (143). In the absence of oxygen, Hx accumulates in body fluids due to hypoxic degradation of adenosine monophosphate (AMP). Studies have shown that vitreous humor Hx levels are elevated in SIDS compared to sudden accidental deaths in infants and children. This suggests that SIDS is preceded by repeated episodes of respiratory failure and hypoxia (17, 18, 144, 145). Animal studies have demonstrated that intermittent periods of hypoxemia result in higher levels of Hx in vitreous humor than chronic hypoxemia (146). In SIDS, a correlation between elevated β-endorphin immunoreactivity in cerebrospinal fluid and increased levels of Hx in the vitreous humor has been reported (147). The neuropeptide β-endorphin is an agonist of opioid receptors in the brain and has the ability to induce respiratory depression. Increased levels may thus aggravate the development of severe hypoxia prior to death in SIDS (147).

Another marker of hypoxia is vascular endothelial growth factor (VEGF). VEGF is highly sensitive to changes in tissue oxygen levels; increased VEGF expression occurs during hypoxia through binding of HIF-1α to a hypoxic responsive element in the VEGF gene (148). A study of VEGF in SIDS found a significantly higher VEGF level in cerebrospinal fluid from cases compared to controls, indicating hypoxia prior to death (149). This finding suggests that the hypoxic episodes occur several hours before death, as this would be the minimum time required for genomic transcription, expression of VEGF and subsequent release into body fluids.

Findings in the brain stem, including gliosis, apoptosis, and reactive astrocytes, also suggest that hypoxemia and episodic hypoxia precedes death in SIDS (150–153). A recent comprehensive study questions the validity of astrogliosis being a feature of SIDS studied glial fibrillary acidic protein (GFAP) expression in astrocytes in tissue from various regions of the brain (154). The findings indicate that GFAP density is high in specific regions of the brain in the first 2 months of life, followed by a decrease during the first year of postnatal development. This was seen in both SIDS and controls. However, the control group were infants who had died of severe infections or cardiovascular disease, conditions that are associated with hypoxia. Thus, what to consider a normal expression of GFAP remains to be clarified.

Neuronal expression of mitochondrial-associated protein-2 (MAP2) is a marker for neuronal damage. It has been shown that when comparing SIDS and controls with and without hypoxic/ischemic injury the level of MAP2-negative reactive neurons was the same in SIDS as in the hypoxic control group (155).

Another key protein in this context is β-amyloid precursor protein (β-APP). When neurons are damaged, the production of β-APP is rapidly upregulated. Similar pattern of β-APP expression have been observed in infants who died from SIDS and infants who died from mechanical asphyxia. Furthermore, it was found higher β-APP staining scores in SIDS sleeping alone compared to SIDS dying when bed-sharing (156, 157). Thus, repeated episodes of hypoxia appear to be a crucial part of the death mechanism in SIDS, leading to neuronal damage and eventually a dysfunction of the serotonergic network.

### 3.7 The serotonergic network

Serotonin (5-HT), often referred to as the guardian of stable breathing, plays a crucial role in numerous physiological processes. This include recovery from hypoxic reflex-apnea and gasping, restoration of heart rate and blood pressure, termination of apnea, restoration of eupnoea, and arousal. Several studies have presented evidence suggesting a disturbed serotonergic network in cases of SIDS, indicating a failure of normal protective response against life-threatening challenges in these cases (26, 42, 43).

Numerous findings in the brainstem support the hypothesis of a serotonergic dysfunction. These includes a reduced serotonergic 5-HT_1A_ and 5-HT_2A_ receptor binding and immunoreactivity (43, 158, 159), a higher 5-HT neuron count and decreased 5-HT_1A_ and 5-HT_2A_ receptor immunoreactivity (160, 161), as well as a lower ratio of serotonin transporter (5-HTT) binding density to 5-HT neuron count (43, 162). Additionally, there is evidence suggesting abnormal 5-HT_2A/C_ and 5-HT_1A_ signaling across multiple medullary nuclei that are vital for arousal and autoresuscitation in at least a subset of SIDS (163). A study of 5-HT and its primary metabolite 5-hydroxy indoleacetic acid (5-HIAA) in medulla from SIDS found lower 5-HT levels in cases compared to controls, indicating a deficiency in medullary serotonergic activity in SIDS (42). Transgenic mice with over-expression of 5-HT_1A_ in serotonergic neurons exhibit sporadic bradycardia and hypothermia that frequently progress to death (164). This indicates that excessive serotonin autoinhibition could be a risk factor for autonomic dysregulation and thus provide a mechanism for the altered serotonin homeostasis in SIDS.

In addition to 5-HTT, which mediates 5-HT reuptake in the synapsis, brain 5-HT metabolism is also influenced by monoamine oxidase A (MAOA), the latter being responsible for intracellular degradation of 5-HT. The dopaminergic activity is closely modulated by the serotonergic system. A study of dopaminergic neurons in midbrain samples from SIDS and controls reported signs of anatomical and functional degenerations of dopaminergic neurons in a large proportion of the SIDS cases compared to deaths from explained causes (165).

Several studies have investigated genetic variation in genes involved in the serotonergic network, including the genes encoding 5-HTT, MAOA and dopamine transporter (DAT) (53, 54, 166–168). It has been reported significant findings, but as several papers not take into consideration that the MAOA gene is located on the X chromosome and do not divide cases and controls according to sex, the results are inconclusive and difficult to interpret (166, 167).

The γ-aminobutyric acid (GABA) is another essential neurotransmitter in the brainstem. GABA neurons in the medulla oblongata regulate homeostasis through interactions with the medullary serotonergic network, and GABA_A_ receptors are critical markers of GABAergic function. It is interesting that in SIDS it is reported reduced GABA_A_ receptor binding density in nuclei in the medullary serotonergic system (169).

Peripherally produced cytokines can cross the blood-brain barrier and bind to cytokine receptors on neurons in the brainstem, influencing sickness behavior, blunt arousal and also suppress respiration (110, 170). IL-6 affects the central nervous system by activating the hypothalamo-pituitary-adrenocortical axis, thereby increasing brain tryptophan and serotonin metabolism. A study of the expression of IL-6 receptors in the brainstem in SIDS found that the mean IL-6R intensity was significantly higher in SIDS than in controls (37). Hence, aberrant interactions between IL-6 and CO_2_-sensing regions in the brainstem may contribute to impaired responses to hypercapnia generated by infection combined with prone sleeping position and rebreathing.

Maternal smoking is a recognized major risk factor for SIDS. Nicotine, the primary component in cigarette smoke, has a direct effect on the serotonergic system. Studies on SIDS have reported an effect of both pre- and post-natal maternal smoking in medullary nuclei containing serotonergic neurons (159, 171). Animal studies have demonstrated that prenatal nicotine exposure induces a significant increase in 5-HT turnover. In 5-HT deficient rat pups, it results in impaired arousal (172, 173). Thus, when nicotine exposure interacts with a mild serotonergic deficiency, autoresuscitation failure may be aggravated.

Collectively, these findings have contributed to the formulation of the “Brainstem hypothesis in SIDS” (26). This hypothesis suggests that at least a subset of SIDS is due to abnormal regulation of the serotonergic system and/or impairments in arousal mechanisms. These abnormalities hinder the infant's ability to respond effectively to common sleep-related stressors, such as hypoxia, hypercapnia, asphyxia, and hyperthermia. One potential scenario involves an increased number of serotonergic neurons which could lead to an excess of extracellular 5-HT. This excess will trigger a compensatory down-regulation of serotonergic receptors (43). Cytokines may constitute a link between a dysregulated peripheral immune system and the brainstem serotonergic network (37).

### 3.8 Aquaporin dysregulation and brain swelling

The aquaporin (AQP) family consists of 13 proteins sharing a common structure that facilitate diffusion of water across cell membranes. AQPs are ubiquitously distributed through the organs, including the brain and central nervous system. The most important water channel in the brain is AQP4, but also AQP1 and AQP9 have been reported present (174, 175).

AQP4 plays a crucial role in maintaining brain water homeostasis, neural signal transduction, and development of brain edema (176, 177). AQP4 is co-expressed with the inwardly rectifying potassium channel Kir4.1, forming a multifunctional unit responsible for the clearance of potassium and/or water following neuronal activity (178). Even minor alterations in the expression or function of the AQP4/Kir4.1 complex can disrupt water/ion homeostasis, affecting brain development, increase susceptibility to seizures and contribute to brain swelling and edema formation. This may be particularly unfavorable during the vulnerable developmental stage in the first months of life.

AQP4 also plays a significant role in infection. Studies have demonstrated that IL-1β, IL-6 and TNFα can upregulate the AQP4 expression in astrocytes (179–181). IL-1β has been shown to stimulate the expression of AQP4 in cerebral tissue in a dose-dependent manner. Both AQP4 mRNA and protein levels are increasing with higher doses of IL-1β (182). Furthermore, TNFα and IL-6 secretion is found reduced in astrocyte cultures from AQP4-knockout mice, providing further evidence of a connection between AQP4 water permeability and cytokine release (181). Thus, it is possible that in some SIDS cases, excessive production of cytokines may contribute to a disturbed water/ion homeostasis and development of brain swelling and edema.

Interestingly, AQP4 expression in the hippocampus is lower in infants with the AQP4 genotype rs2075575 CT/TT than the CC genotype, and higher in the youngest infants (≤12 weeks) (183). High-water content of brains of the youngest infants, and finding of association between the CT/TT genotype and the brain/body weight ratio in SIDS victims younger than 12 weeks, may indicate that the rs2075575 CT/TT genotype represents a genetic risk factor for a subgroup of SIDS (56). One possible underlying mechanism is an increased risk of subclinical seizures and a decreased ability to eliminate hypoxia-generated edema fluid.

The aquaporins are important also with regard to the serotonergic network, and it is shown that lack of AQP4 expression is paralleled by alteration in the levels of 5-HT in various areas of the brain (184). It has also been shown that AQP4 participates in regulating K^+^-stimulated releases of neurotransmitters, including 5-HT (185). This indicates that maintaining a well-functioning water balance is important also for the regulation of neurotransmitters, suggesting a possible connection between the expression and function of aquaporins and the serotonergic imbalances observed in SIDS.

Another common aspect in SIDS is oxidative stress and hypoxia prior to death (17, 18, 144, 145, 149). One key molecule in the oxygen-sensing system is the transcriptional regulator hypoxia-inducible factor (HIF), which controls a range of oxygen responsive target genes such as VEGF and erythropoietin. VEGF has been found upregulated in SIDS, and both VEGF and HIF-1α have the ability to up-regulate the expression of AQP4 (149, 186–188). This up-regulation may serve as a protective response to hypoxia by facilitating elimination of hypoxia-initiated brain edema. However, the upregulation may also induce a molecular pathway leading to upregulation of AQP4, resulting in brain swelling and edema formation through disruption of the blood-brain barrier (187, 188). Furthermore, it is shown that hypercapnia-induced IL-1β overproduction, combined with hypoxia, may increase blood-brain barrier permeability (189).

A few studies have reported higher brain weight in SIDS cases compared to both controls and established standards (190–192). Macroscopic signs of cerebral edema has been described in cases of SIDS, even though these findings are inaccurate. It is important to consider that increased fluid content in postmortal brain tissue might represent congestion associated with the death process, rather than vital edema that developed earlier (193, 194). The brain undergoes significant growth in both size and weight during the first year of life, and one study has indicated an association between specific variants in the AQP4 gene and enlarged brain/body weight ratio in SIDS cases under 3 months of age (56). It is hypothesized that the observed increased brain weight in SIDS, when controlled for age and body weight, may reflect abnormal cerebral development potentially impairing neural control (192).

## 4 Concluding remarks

The vicious spiral is a model suggesting a final common pathway in a large proportion of SIDS (Figure 2). The initiating event is a synergistic effect of trigger events, i.e., prone position with face down in a soft mattress, combined with slight infection/common cold and hot environment, that induce hypercapnia. An adverse response of the mucosal immune system may, via either retrograde axonal transport or blood born, induce cytokine production in the cerebrospinal fluid. The subsequent increase in IL-6 levels induces fever, resulting in thermal stress and hyperthermia. These conditions can cause bradycardia and irregular breathing, leading to ineffective gasping with impaired autoresuscitation. This sequence of events culminates in hypoxemia and downregulation of respiration, further exacerbated by IL-1β and nicotine exposure. Next, hypoxic markers such as hypoxanthine and VEGF are expressed. If the serotonergic network fails to induce an appropriate compensatory mechanism, the infant enters a state of severe hypoxia. This impaired response of the serotonergic network may be worsened by a disturbed water homeostasis in the brain, inducing brain swelling and ultimately coma and death.

The critical step in this model is the downregulation of respiration, followed by impaired autoresuscitation and the accumulation of hypoxic markers. In an infant without vulnerabilities, hypercapnia and hypoxia induce protective responses in the brainstem, ultimately restoring normal oxygen levels. However, in an infant susceptible to SIDS due to a combination of a vulnerable developmental stage and/or having a genetic predisposition, these protective mechanisms are impaired, leading to the progression of hypoxia, subsequent brain swelling, and eventually death.

Understanding the etiology and pathogenesis of SIDS is crucial for preventing these deaths. Prevention of SIDS requires increased knowledge of each step in the vicious spiral, beginning with identifying and avoiding external risk factors. Equally important is a comprehensive understanding of genetic and developmental vulnerabilities, as well as further examination of the mechanisms that, when combined, lead to the death of an infant.

## 5 Limitations

SIDS is a devastating but rare event, and the SIDS rate have steadily decreased since the back-to-sleep campaigns in the early 1990s. This trend is seen reflected in reduction of total post neonatal mortality (8, 81). Because of this very positive reduction, many published papers report relatively few SIDS cases and even fewer suitable controls. Ideally, the most appropriate control group for SIDS research would consist of healthy infants who experience a sudden and rapid death from a violent cause and yet have an uninjured brain. Fortunately, such cases are rare.

The impact of correct controls, as well as the impact of postmortem time and temperature, might be of particular importance with regard to hypoxic markers. Though there are morphological clues for previous episodes of hypoxia in SIDS, it has been difficult to find markers for demonstration of asphyxia in the period preceding death (10). Our study of increased levels of Hx in vitreous humor in SIDS was commented on by Beckwith, who pointed to uncertain postmortem intervals and the “warm room effect” peculiar for SIDS (17, 18, 195). A study from Carpenter et al, which by comparing Hx level in cerebrospinal fluid in SIDS and deaths from a “variety of conditions” concluded that Hx could not be a marker of pre-mortem hypoxia in SIDS (196). The latter study illustrates the challenges posed by lack of appropriate controls in SIDS research. In our opinion, the only suitable controls are infants and small children who have died suddenly from accidents (197). By controlling for postmortem time and ambient temperature, we were able to confirm previous observations of significant higher Hx levels in SIDS than in violent death, whether there were no significant difference between SIDS and infectious death (144, 145). Nevertheless, it is important that these and other findings are confirmed in independent studies.

Although the definition of SIDS always has involved aspects of exclusion, the diagnostic criteria have evolved over the years. This evolution began with the Seattle definition in 1969, continued with the NICHD definition in 1989, and eventually led to the now most widely used San Diego definition in 2004 (1–3). Despite these advancements, a large proportion of SIDS research include infant cohorts without clear specification of definition used or the extent of the investigations performed (6, 198, 199). This lack of standardization complicates the ability to compare data across studies.

As stated for studies of postmortem asphyxia, a limitation of today's research on death mechanisms in SIDS is lack of reproducing studies. Several factors contribute to this, including limited access to tissue and fluid samples from SIDS cases classified according to international standards, suitable controls, and expertise and facilities to perform high quality studies (6, 200). To address this issue, largescale collaborative studies that include well defined SIDS cases and control groups are crucial. These studies should aim to systematically replicate and validate previous findings. Such initiatives should be encouraged.
